# Variability of *Mycobacterium avium* Complex Isolates Drug Susceptibility Testing by Broth Microdilution

**DOI:** 10.3390/antibiotics11121756

**Published:** 2022-12-04

**Authors:** Danila Zimenkov

**Affiliations:** Center for Precision Genome Editing and Genetic Technologies for Biomedicine, Engelhardt Institute of Molecular Biology, Russian Academy of Sciences, 119991 Moscow, Russia; z@biochip.ru; Tel.: +7-(916)-546-9782

**Keywords:** nontuberculous mycobacteria, avium, intracellular, MAC complex, resistance, MIC, macrolides, amikacin, ethambutol

## Abstract

Non-tuberculous mycobacteria are widely distributed in environments and are capable of infecting humans, particularly those with a compromised immune system. The most prevalent species that cause nontuberculous mycobacterial lung diseases are slow-growing bacteria from the *Mycobacterium avium* complex (MAC), mainly *M. avium* or *M. intracellulare*. The key treatment of MAC infections includes macrolides, ethambutol, and rifampicin; however, the therapy outcomes are unsatisfactory. Phenotypic drug susceptibility testing is a conditional recommendation prior to treatment, and critical concentrations for clarithromycin, amikacin, moxifloxacin, and linezolid have been established. In this review, data from studies on the determination of MIC of clinical isolates using the broth microdilution method were summarized. A significant variation in the MIC distributions from different studies was found. The main reasons could impact the findings: insufficient reproducibility of the phenotypic testing and variation in species lineages identified in different laboratories, which could have various intrinsic susceptibility to drugs. For most of the drugs analyzed, the MICs are too high, which could undermine the treatment efficiency. Further improvement of treatment outcomes demands the validation of microbiological resistance criteria together with the identification of molecular mechanisms of resistance.

## 1. Introduction

The genus *Mycobacterium* contains about 200 species, of which the best known are *M. tuberculosis* and *M. leprae* [1]. All other mycobacteria, called nontuberculous mycobacteria (NTM), are considered conditionally pathogenic to humans and capable of causing generalized disease under certain conditions, particularly in immunocompromised individuals. Studies in North America, Europe, and Asia have shown an increased incidence of NTM in recent decades [2,3,4]. The estimated prevalence of NTM increased from 2.4 cases per 100,000 in the early 1980s to 15.2 cases per 100,000 in 2013 in the United States [5], in Canada the incidence in 2010 was as high as 20–25 cases per 100,000 population [6]. In South Korea, NTM disease increased from 9.4 in 2009 to 36.1 in 2016 cases per 100,000 population [7]. The incidence of pulmonary disease caused by NTM ranged from 1.07 in French Guiana in 2018 [2] to 4.73 in 2015 in the USA [8]. The annual prevalence in the latter study was estimated to be 11.7 per 100,000 which is caused by the long duration of the disease. A lower prevalence of 4.5 was recorded in 2019 in The Netherlands [9], which is close to the estimated rate of 4.8 in South Korea in 2016 [4]. 

The rates of pulmonary NTM disease increase dramatically with age: the incidence and prevalence in people aged 65 and older were equal to 18.37 and 47.48, respectively [8]. Furthermore, certain groups of people are predisposed to the development of NTM disease. These include patients with genetic or acquired structural lung diseases such as cystic fibrosis, chronic obstructive pulmonary disease, bronchiectasis, alpha-1-antitrypsin deficiency, tuberculosis and lung cancer, pulmonary fibrosis, and pneumoconiosis [10]. Patients with immunosuppression due to primary [11] and acquired immunodeficiency syndromes that accompany HIV infection and hematologic malignancies, and patients receiving systemic glucocorticosteroid or cytostatic therapy are also susceptible to NTM infection [12]. The clinical significance of NTM detection in the verification of infectious complications in cardiothoracic and aesthetic surgery has been observed [13].

The range of species detected in clinical studies differs markedly from country to country and region to region, often due to laboratory diagnostic capabilities. However, mycobacterial diseases are most often caused by the slow-growing species of MAC complex—*M. avium* ssp. and *M. intracellulare* ssp. or by fast-growing species of Chelonae-Abscessus group (ABS)—*M. abscessus* ssp., *M. chelonae* and *M. immunogenum*. Mycobacteria of the MAC complex dominate in almost all published studies, taking about 50% of all isolates [14].

The natural habitats of NTM are water and soil, and the main route of pulmonary infections is the inhalation of aerosols and dust [15]. Water supply chains such as shower heads, plumbs, and heater-cooler devices were traced as the sources of clinical isolates [16,17]. The possible transmission of the MAC strain from human to human was proposed since clusters of genetically close clinical isolates obtained from different patients were identified [18]. However, the case of transmission is hardly distinguished from the acquisition of the strain from the same source. 

The pathogenesis of pulmonary diseases caused by nontuberculous mycobacteria is similar to that of tuberculosis, but the therapeutic regimens used in tuberculosis therapy cannot be tolerated due to the natural resistance of mycobacteria to most drugs [19].

Macrolides, rifampicin, and ethambutol are currently recommended by international respiratory medicine and infectious diseases societies for the treatment of MAC infections; in the case of resistance to macrolides, moxifloxacin or isoniazid are included in the regimen. The use of injectable amikacin or streptomycin [20], which can cause serious complications, especially with prolonged therapy, is also offered at the discretion of the physician in severe cases. The percentage of positive therapy outcomes is currently recognized as unsatisfactory, and a significant number of patients do not achieve sputum conversion within 12 months of therapy [21]. This is particularly important in macrolide-resistant mycobacterial diseases with conversion rates of 15–36% [21]. Continuation of antibiotic therapy for 12 months after sputum conversion is currently recommended to improve the rate of positive outcomes [20,22]. However, a recurrent disease develops in 33% of patients [23].

Clarithromycin and amikacin resistance testing is recommended when prescribing treatment of MAC infection, and a broader panel of drugs should be tested for the macrolide-resistant cases. However, the phenotypic data from the isolates should ‘guide, but not dictate, treatment regimens’ [20].

The microtiter plate method for MIC determination is now widely used, as recommended by CLSI [24]. The use of Sensititre SLOMYCO and RAPMYCO plates with pre-diluted and lyophilized drugs [25] is particularly convenient in routine laboratory practice. Although criteria have been developed to determine the resistance of MAC isolates to clarithromycin, moxifloxacin, amikacin, and linezolid [24], it should be noted that the low success rate of drug therapy most likely requires the rethinking of the results of the pathogen phenotypic analysis, their relationship with pharmacokinetic data, and finally, the analysis of clinical outcomes. The question of the reproducibility of the phenotypic results of non-tuberculous mycobacteria has already been raised, and the results of parallel testing of typical strains have been found to be unsatisfactory [26]. In this regard, it is of particular interest to compare the resistance spectra of clinical mycobacterial isolates performed in different regions of the world, since these data are used both to adjust therapy and to develop or validate criteria for phenotypic resistance detection.

## 2. The Studies Included in the Review

Relevant publications were searched with PubMed and Google. Some articles were identified during a review on related topics and were not found using queries such as ‘minimal inhibiting concentration AND avium’.

The publications were analyzed for sample type, the phenotypic method used, and availability of raw data. Studies performed using a solid medium and the radiometric Bactec 460 method were excluded. A large percentage of studies that reported only population-based MIC_50_ and MIC_90_ or binary resistant/susceptible data based on the currently approved criteria were also excluded from the review. Some studies reported only a graphical distribution of MICs without providing the exact number of isolates. In this case, the data were obtained by parsing histograms using the vector graphics editor. The resulting small error in the number of isolates did not exceed 0.3%. The final list of studies included in the review is presented in Table 1. Characteristics of all studies analyzed are given in the Supplementary Materials (Table S1). EUCAST MIC data for amikacin, clarithromycin, ethambutol, linezolid, moxifloxacin, rifampicin, rifabutin, and trimethoprim-sulfamethoxazole for *M. avium* and *M. intracellulare* were added to the analysis.

For two studies [30,34], the strains with the two highest MIC values were combined into one category, which was due to the technical aspect of the processing of the results. The MIC distributions of the isolates given below are presented on a synthetic scale: the distributions were normalized to values between 1 and 4, proportional to the maximum value in the distribution. Therefore, it became possible to compare studies with significantly different numbers of isolates in one graph.

## 3. Antibacterial Drugs

### 3.1. Macrolides

Macrolides are a key drug in the therapy of MAC infections [20,22]. For clarithromycin, CLSI has approved the criteria for the interpretation of MICs obtained by the broth microdilution method: strains with MIC ≤ 8 mg/L are classified as susceptible, strains with MIC = 16 mg/L—intermediate, and with MIC ≥ 32 mg/L—resistant to macrolides [24]. For strains in the intermediate category, a follow-up monitoring of MIC levels is considered necessary, as such levels may indicate the development of resistance.

**Figure 1:**
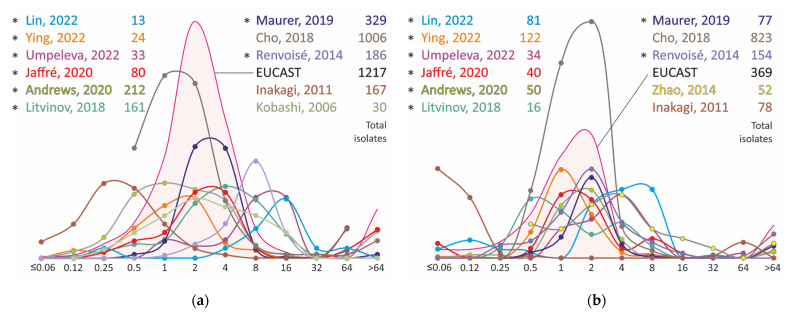
Clarithromycin MIC distribution for clinical isolates of *M. avium* (**a**) and *M. intracellulare* (**b**). * MIC obtained using Sensititre SLOMYCO plates.

The clarithromycin MIC data for 3458 strains of *M. avium* and 1896 strains of *M. intracellulare* species were analyzed in total (Figure 1). *The M. avium* isolates are characterized by a wide variety of resistance spectra with a range of distribution modes from 0.25 to 16 mg/L. The distributions of MICs obtained using SLOMYCO plates form a more compact group with modes ranging from 2 to 16 mg/L. Discrepancies in the results may be due to problems with the MIC measurement method, namely, the dependence on the pH of the medium [43]. An alternative explanation could be the heterogeneity of the *M. avium* population and the heterogeneity of the phenotypic characteristics of isolates in different regions. The better convergence of clarithromycin MIC test results for *M. intracellulare* strains may support this assumption (Figure 1). All distributions can be classified into three groups with maximums of 1 or 2 mg/L and a broadened distribution with maximums of 4–8 mg/L. The results of two studies, Inagaki, 2011 [40] and Litvinov, 2018 [34], differ noticeably from the others. In the former, only extreme values of clarithromycin MICs were found, which can be attributed both to the peculiarities of strain sampling and to technical measurement errors. In the latter, the observed bimodal distribution with peaks at 0.5 and 4 mg/L may be due to the small number of strains taken into the study.

In most studies, the distribution increases at MIC values 64 mg/L and this resistance is associated with mutations at positions 2058 and 2059 of the *rrl* gene that encodes the 23S rRNA [44,45]. Many studies have reported high (>90%) sensitivity of resistance detection by analysis of these mutations [40,46]. Furthermore, the commercial molecular test system Hain Lifescience GenoType NTM-DR, capable of detecting mutations at positions 2058–2059 of the *rrl* gene, also has a high diagnostic performance to detect resistant strains [47,48]. However, in the Christianson study, low specificity was reported, which was due to the phenomenon of mixed populations: in about half of the resistant *M. avium* isolates mutations were detected by sequencing only after additional cultivation with the drug in vitro [46]. 

The therapeutic efficacy of macrolides can be attributed to the increased concentration of the drug in macrophages and lung lesions compared to plasma [49] and to the synergistic action with other drugs [50]. On the other hand, clarithromycin does not have bactericidal action against resting forms, and concomitant administration of rifampicin or rifabutin dramatically reduces plasma concentrations of clarithromycin [51]. The immunomodulatory properties of macrolides are also expected [52], but the lack of clinical effects of macrolide use in macrolide-resistant diseases does not support this assumption [53].

On the basis of MIC data, it could be concluded that isolates of the MAC complex have a reduced susceptibility to macrolides at least, which is due to unknown determinants [54]. They lack a major resistance gene found in a wide spectrum of infectious agents, the *erm* gene, which encodes the rRNA methyltransferase, responsible for inducible resistance to macrolides in strains of the *Mycobacterium abscessus* complex [55] and resistance of *M. tuberculosis* [56]. Other possible mechanisms found in other bacterial species include substitutions in the ribosomal proteins L4 and L22 [57,58], drug export [59], and reduced cell wall permeability [60].

### 3.2. Ethambutol

Ethambutol is a first-line drug for the therapy of susceptible forms of tuberculosis. Despite its lack of sterilizing activity and its effect only on the growing forms of the pathogen, ethambutol has effectively replaced streptomycin in therapy regimens due to comparable efficacy and lower toxicity [61]. For nontuberculous mycobacteria, there are currently no established criteria for determining resistance, and there are no recommendations for adjusting therapy if a high MIC is detected.

**Figure 2:**
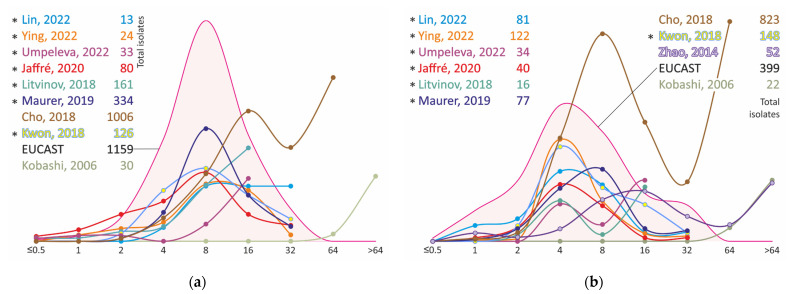
Ethambutol MIC distribution for clinical isolates of *M. avium* (**a**) and *M. intracellulare* (**b**). * MIC obtained using Sensititre SLOMYCO plates.

The MIC distributions of *M. avium* strains of ethambutol converge significantly better than the MIC distributions of clarithromycin. In almost all studies they are bell-shaped curves with a mode of 8 mg/L (Figure 2, Table S3). Distributions with a restricted range may have an increase in the upper bond value because they include all strains with an MIC greater than or equal to the previous one. Thus, strains in the Cho, 2018 [37] distribution plotted at MIC = 64 mg/L in the graph (Figure 2A) have MIC ≥ 32 mg/L, and the distribution itself may also be bell-shaped.

However, in the case of *M. intracellulare*, the same considerations about the same study by Cho, 2018 [37] are probably no longer applicable, because the number of strains with MIC ≥ 32 mg/L is too high and two subpopulations of strains possessing different MICs in vitro can be assumed. Furthermore, the bimodal distribution was also found in Umpeleva, 2022 [30], Litvinov, 2018 [20], and Zhao, 2014 [39]. Most studies show the first mode of distribution at 4 mg/L, which is slightly lower compared to *M. avium*.

The activity of ethambutol, its mechanisms of resistance, and clinical efficacy have been thoroughly investigated in the application to tuberculosis therapy. The MIC distribution of ethambutol-resistant and *embB* gene mutations bearing clinical strains of *M. tuberculosis* partially overlaps with susceptible strains, and has a maximum distribution at a value of 8 mg/L [62]. Most resistant strains have a MIC from 4 to 16 mg/L, which almost coincides with the MIC distributions for presumably susceptible *M. avium* and *M. intracellulare* isolates. In tuberculosis therapy, a critical concentration of 5 mg/L is accepted to determine resistance to ethambutol in a liquid medium [63]. This concentration is lower than the maximum concentration achieved in plasma at standard doses; for example, the average maximum concentration was only 3.3 mg/L at a dose of 19.5 mg/kg [64]. However, ethambutol has been shown to accumulate in lung tissue and cavities, exceeding the necessary thresholds of action [61].

There are several important aspects with respect to the clinical relevance of MIC measurement of ethambutol for the treatment of nontuberculous mycobacterial infections. First, in a clinical study of 37 patients with clarithromycin-resistant MAC infection, ethambutol significantly increased sputum conversion rates compared to rifampicin, streptomycin, or fluoroquinolones [53]. Clarithromycin did not have an effect in this study, as expected. Second, the use of ethambutol in macrolide-sensitive MAC infections is believed to reduce the rate of resistance development [65,66].

Currently, there is no consensus on the correlation between the MIC of ethambutol and the clinical outcome of therapy. Adachi et al. believe that there is no such correlation, but only a small sample of cases has been studied, ranging from 1 to 3 isolates with each MIC [53]. In contrast, in the study by Kwon et al., there is a notable negative correlation between favorable outcome rates with the MIC of rifampicin and ethambutol [35]. This study included 274 cases of mycobacterial diseases caused by *M. avium* and *M. intracellulare*, and therapy outcomes were evaluated for at least several dozen cases with each MIC of the pathogen.

### 3.3. Rifampicin

Rifampicin is the most effective first-line drug for the treatment of susceptible tuberculosis introduced in the 1960s. Its mechanism of action and resistance mechanisms in *M. tuberculosis* has been extensively investigated; however, the critical concentration used for decades has recently been reduced from 1.0 mg/L to 0.5 mg/L [67], resulting in the reclassification of some susceptible strains as resistant.

**Figure 3:**
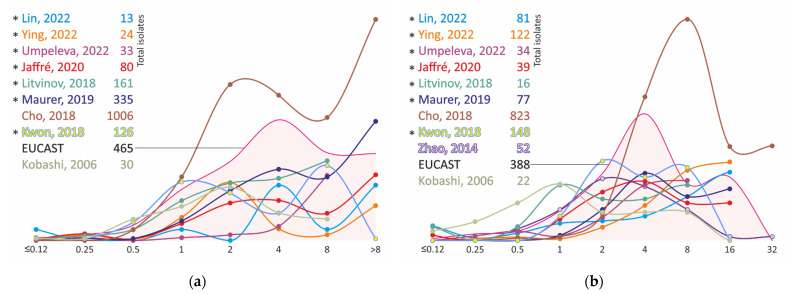
MIC distribution of rifampicin for clinical isolates of *M. avium* (**a**) and *M. intracellulare* (**b**). * MIC obtained using Sensititre SLOMYCO plates.

The MIC distributions of clinical strains of *M. avium* and *M. intracellulare*, even obtained by the same method on SLOMYCO plates, have significant differences. Most distributions have two modes, one in the range of 2–4 mg/L and the other in the range of 8 mg/L or higher (Figure 3, Table S4). Apparently, despite the absence of an *arr* locus that leads to resistance to rifampicin in most nontuberculous mycobacteria, other determinants could be responsible for the reduced susceptibility and variability in MIC data of MAC isolates.

The role of rifampicin in the therapy of MAC infections is controversial [68,69], rifampicin is markedly less effective compared to macrolides and ethambutol, and may be used as a third drug in complex therapy. In the Kwon study [35], there was a marked correlation between the drop in the percentage of favorable outcomes depending on the MIC of rifampicin as for ethambutol. On the other hand, co-administration of rifampicin or rifabutin dramatically lowers the plasma concentration of clarithromycin [51], as mentioned above.

A possible alternative to rifampicin is the semi-synthetic drug rifabutin, which is also present on the SLOMYCO panel for MIC testing. A total of 641 strains of *M. avium* and 370 strains of *M. intracellulare* were analyzed in six published studies and EUCAST data. Three more studies had data on MAC isolates without species determination (Table S5). Most *M. avium* strains had MICs ≤ 0.25 mg/L (*n* = 378, 59%). One study for 33 strains [30] had a distinct distribution, with 23 strains having MIC ≥ 8 mg/L. For *M. intracellulare* strains rifabutin MIC distributions are shifted to higher values, strains with a minimally detectable MIC ≤ 0.25 mg/L accounting for 31% (*n* = 113 of 370) (Table S5).

The benefits of rifabutin in the form of higher tissue concentrations and lower MICs compared to rifampicin are diminished by higher levels of side effects [70]. Furthermore, a meta-study of clinical data did not show an increased efficacy of rifabutin compared to rifampicin in the therapy of MAC infections [71].

### 3.4. Fluoroquinolones

Fluoroquinolones are widely used in drug-resistant tuberculosis therapy regimens. *M. tuberculosis* resistance to fluoroquinolones is associated with the emergence of substitutions in the A and B subunits of DNA gyrase. The epidemiological threshold ECOFF is estimated to be 0.5 mg/L, and the currently approved clinical breakpoint is 2 mg/L [63].

**Figure 4:**
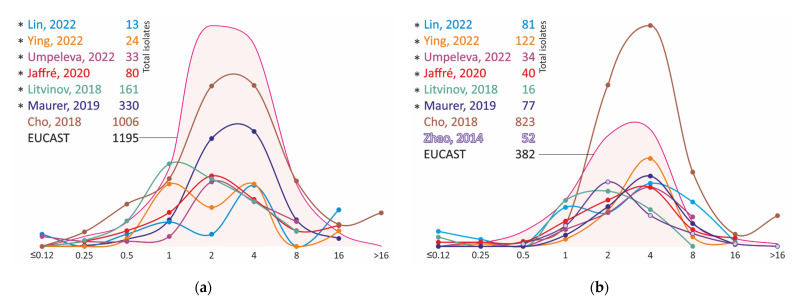
Moxifloxacin MIC distribution for clinical isolates of *M. avium* (**a**) and *M. intracellulare* (**b**). * MIC obtained using Sensititre SLOMYCO plates.

**Figure 5:**
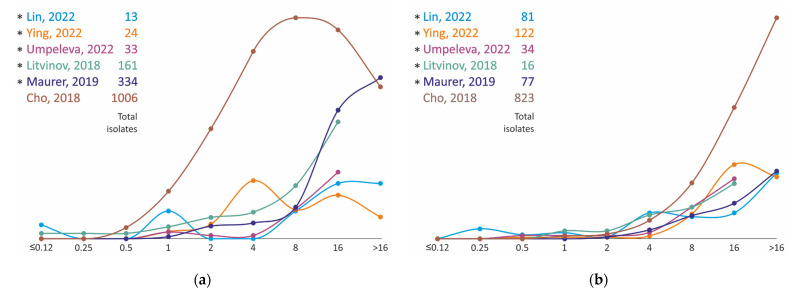
Ciprofloxacin MIC distribution for clinical isolates of *M. avium* (**a**) and *M. intracellulare* (**b**). * MIC obtained using Sensititre SLOMYCO plates.

The MIC distributions of moxifloxacin isolates of MAC vary less than the results obtained for clarithromycin, and, in general, the main number of strains have MIC of moxifloxacin in the range of 1 to 8 mg/L (Figure 4, Table S6), which is comparable to the MICs of fluoroquinolone-resistant strains of *M. tuberculosis.* The strains show even higher levels of resistance to ciprofloxacin (Figure 5, Table S7).

For MAC isolates, CLSI recommends two breakpoint moxifloxacin concentrations of 1 and 2 mg/L [24]; strains with MIC = 2 mg/L are classified as intermediately resistant. Only studies by Lin, 2022 [28] and Ying, 2022 [29] with bimodal MIC distributions meet these criteria, but the number of strains tested is small. An attempt to detect mutations in the *gyrA* and *gyrB* genes in strains presumably resistant to moxifloxacin using CLSI criteria was unsuccessful [72].

Moxifloxacin can accumulate within macrophages at concentrations 6–7 times higher than plasma concentrations, but it has no effect on intracellular forms [73], apparently due to the pathogen being in dormant state with dramatically reduced replication and division rates. Assessment of the critical concentration of moxifloxacin at 0.25 mg/L, obtained in the hollow-fiber *M. avium* model, and the high natural MIC of clinical strains exclude the possibility of its effective use in therapy [73]. Fluoroquinolones have not been shown to accelerate sputum conversion in macrolide-resistant MAC infections [54,74], do not prevent the development of resistance to macrolides, and, in general, are not currently recommended for the treatment of MAC infections [65].

### 3.5. Isoniazid

Isoniazid, along with rifampicin, is one of the main drugs for the treatment of tuberculosis. Isoniazid is activated in the cell by KatG catalase-peroxidase, and the differences in substrate specificity of this enzyme explain the differences in sensitivity to isoniazid of different mycobacterial species [75]. The critical concentration of isoniazid on liquid media for the *M. tuberculosis* clinical strains is 0.1 mg/L, the clinical breakpoint is 0.4 mg/L [24], the ECOFF of susceptible strains is 0.125 mg/L [76], while the MIC of resistant *M. tuberculosis* strains is greater than or equal to 4 mg/L.

**Figure 6:**
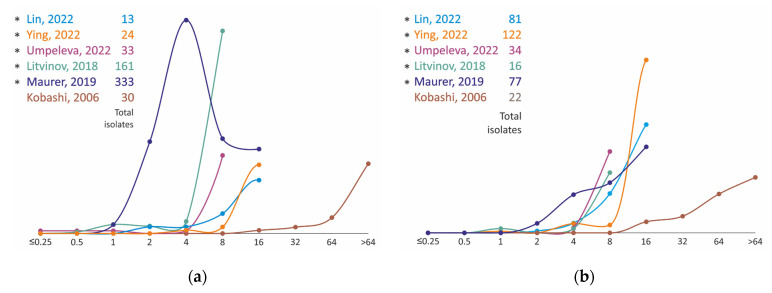
Isoniazid MIC distribution for clinical isolates of *M. avium* (**a**) and *M. intracellulare* (**b**). *—MIC obtained using Sensititre SLOMYCO plates.

Even in early studies, the high MICs of isoniazid of the *M. avium* complex isolates were observed [77]. Analysis of MIC distributions confirmed that *M. avium* and *M. intracellulare* resistance levels are equal to or greater than the MICs of *M. tuberculosis* isoniazid-resistant isolates with mutations at the *katG* and *inhA* loci (Figure 5, Table S8). In most studies, strains have MICs ≥ 8 mg/L, with the exception of the MIC distribution of *M. avium* isolates obtained in the Maurer study [36]. This study reported lower MICs, ranging from 2 to 8 mg/L (Figure 6, Table S8).

Isoniazid is indicated for use in macrolide-resistant MAC infection by the British Thoracic Society with the lowest grade of recommendation D [20]. The use of isoniazid for the therapy of MAC infections has been removed from current recommendations of the American Thoracic Society (ATS) [22] based on numerous observations of ineffectiveness; in an earlier version of 1990, isoniazid was part of therapy regimens [78].

### 3.6. Amikacin

Amikacin, a semi-synthetic broad-spectrum drug from the aminoglycoside group, inhibits protein synthesis by binding to the A-site of the ribosome [79]. For a long time, amikacin was included together with kanamycin and capreomycin in the second-line therapy for tuberculosis. However, due to the high level of side effects, it is currently placed in group C of the WHO recommendations, used only when group A and B drugs cannot be administered [80]. The main mechanism of resistance to amikacin is a 1401 g substitution (corresponds to 1408 position in *E. coli* and *M. avium* numbering) in the 16S rRNA gene, which also leads to cross-resistance to kanamycin and capreomycin [81]. The approved critical concentration of 1 mg/L for clinical *M. tuberculosis* strains has been confirmed in a large number of studies, and the MIC of resistant strains shifts to the range of more than 30 mg/L [63].

Amikacin is considered effective against MAC infections and is approved for the therapy of nontuberculous mycobacterial diseases [20,22,70]. ATS also recommends amikacin susceptibility determination [22], despite a low evidence base of clinical effects [82]. Strains with MIC ≤ 16 mg/L are classified as susceptible, MIC ≥ 64 mg/L as resistant, and strains with MIC = 32 mg/L are assigned to the intermediate resistance category [24]. An increase in the MIC of the pathogen with a 1408 g mutation in the 16S rRNA gene has been observed during failed therapy in the clinic [83]. Other types of substitutions in the peptidyl-transferase center of the ribosome have also been detected in resistant isolates [84].

A liposomal inhaled formulation of amikacin (ALIS) was developed to reduce the side effects of amikacin administration. This drug formulation led to higher concentrations of the drug in lung tissue, while plasma concentrations were 4 times lower compared to intravenous administration. Consequently, the critical resistance concentration for this form of the drug is adjusted to 64 mg/L [24]. Clinical safety and efficacy studies have shown higher sputum conversion rates when ALIS is added to standard therapy [85]. ALIS has also been suggested to be a good alternative to ethambutol in the therapy of MAC infection to prevent the development of resistance [65]. This form of amikacin is currently approved by the FDA for use in difficult-to-treat MAC infections [22].

**Figure 7:**
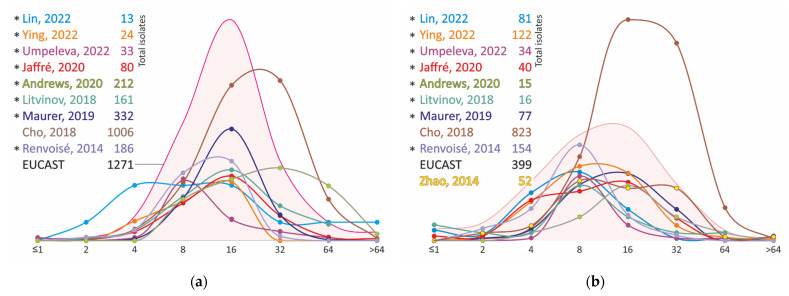
Amikacin MIC distribution for clinical *M. avium* (**a**) and *M. intracellulare* (**b**) isolates. *—MIC obtained using Sensititre SLOMYCO plates.

The analysis of the MIC distribution of *M. avium* isolates for most of the studies performed using SLOMYCO plates converged well (Figure 7, Table S9), with maximums located at 16–32 mg/L, except for one study with a small number (*n* = 13) of isolates [28]. A study of 1006 Cho strains performed according to CLSI standards yields a broader distribution. Similar results were obtained for *M. intracellulare*, although all distributions shifted toward higher MICs within 1 dilution compared to *M. avium*. Interestingly, in an external quality assessment study of European laboratories, the MIC results for amikacin converged worse than those for clarithromycin [26]. However, in this review, the opposite is observed.

### 3.7. Linezolid

Linezolid, also as amikacin, is a drug that acts on the bacterial translational apparatus by binding to the ribosome and is, therefore, widely used in the therapy of various infectious diseases. In tuberculosis therapy, it is among the group A drugs for the treatment of drug-resistant tuberculosis [80]. The critical concentration in liquid media in the Bactec MGIT960 system is 1 mg/L [63]. In the therapy of nontuberculous mycobacterial infections, linezolid is used only in the continuation phase as an adjunctive drug for infections caused by *M. abscessus* [20].

**Figure 8:**
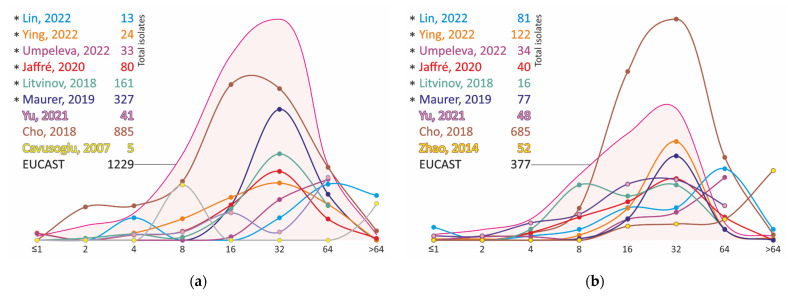
Linezolid MIC distribution for clinical *M. avium* (**a**) and *M. intracellulare* (**b**) isolates. *—MIC obtained using Sensititre SLOMYCO plates.

The analysis of the MIC distributions of clinical *M. avium* and *M. intracellulare* strains also varies greatly from study to study. However, virtually no strains with MIC ≤ 2 mg/L have been reported in all studies; the majority of isolates have MICs in the range of 8 to 64 mg/L (Figure 8, Table S10). In pharmacokinetic/pharmacodynamic studies, the C_max_ of linezolid at the standard 600 mg dosage was 21 ± 6 mg/L [86,87], which limits its applicability if the MIC of the pathogen is ≥ 8 mg/L.

High MICs of linezolid led to studies of the evaluation of next-generation oxazolidinones. For MAC mycobacteria sutezolid MIC distributions are shifted toward lower values: for *M. avium* MICs are in the range from 1 to 8 mg/L; for *M. intracellulare* are in the range from 0.25 to 4 mg/L [31]. In the same study, the MIC distributions of tedizolid and delpazolid were almost identical to those of linezolid. However, in an earlier study, the MIC_50_ and MIC_90_ of tedizolid were significantly lower than those of linezolid [88]. Further studies on the resistance of MAC isolates are needed to establish the feasibility of using linezolid and its analogues in therapy.

### 3.8. Other Drugs

Co-trimoxazole (trimethoprim-sulfamethoxazole) is recommended only for the treatment of *M. abscessus* and *M. simiae* as an adjuvant to the main therapy [20,70]. There are no clinical studies of this drug against MAC infections, with the exception of a retrospective study of the efficacy of preventing MAC infections in HIV-positive patients [89]. The MIC distributions obtained in the different studies, as for most drugs, are not entirely comparable, but most strains have MICs in the range 1/19–4/76 mg/L range (Table S11). Given that the plasma concentration of sulfamethoxazole can reach 161.01 ± 69.154 mg/L [90], it can be assumed that co-trimoxazole may be active against a part of the clinical MAC isolates.

Extremely high MICs that exclude any possibility of their efficiency have been reported for three other drugs presented on the SLOMYCO plate. The MIC distributions of streptomycin, ethionamide, and doxycycline are given in the Supplements (Tables S12–S14).

## 4. Discussion

There are only a few effective drugs against mycobacterial MAC infections with approved resistance criteria [91]. The range of MICs of presumably susceptible isolates is very wide and is hardly comparable between studies, even if the same method is used. This leads to the question of whether susceptible isolates are really susceptible [92]. The problem in determining the resistance of nontuberculous mycobacteria lies both in the poorly studied nature of clinically relevant mycobacterial populations and in the poor reproducibility of the MIC results in different laboratories, as previously shown [26] and also as shown in this review. Studies not included in this study that contain only population MIC_50_ and MIC_90_ parameters also report significant differences in resistance levels.

The parameters of the phenotypic method for determining MIC, such as the microbial medium, in particular its pH value, and the stability of antibacterial substances, which is especially important in long-term cultivations of slow-growing mycobacteria [40], influence the results. The method of plate reading could also be important—the use of resazurin was shown to improve the reproducibility of MIC determination compared to the standard protocol [93]. The genetic variability of isolated strains in different regions of the world cannot be excluded either. Although different species and subspecies in the MAC complex do not have identical MIC_50_ and MIC_90_ values [94], the five lineages of *M. avium* may also differ in the level of intrinsic resistance to antibacterial drugs [95]. 

It should be mentioned that the microbiological characterization of clinical isolates may be difficult due to the presence of a phase variation effect in *M. avium* ssp. experimentally observed as a difference in colony color when stained with Congo red [96]. In clinical specimens, white colonies with elevated levels of antimicrobial resistance are predominantly isolated. The transition in vitro to a stained phenotype is often observed, and the reverse transition appears to be difficult or impossible [97]. 

Another factor that makes it difficult to determine the phenotype of clinical isolates is the phenomenon of heteroresistance, when a mixture of wild-type and mutant strains is found in the same sample [46]. The in vitro MIC of such a mixture could be significantly lower than the MIC of the mutant resistant fraction, but rapid resistance selection will occur during therapy.

The standard pharmacokinetics/pharmacodynamics model of antibacterial drug action takes into account the maximum plasma concentration C_max_ achieved at a given dose, the MIC of the drug against the pathogen in vitro, the time of exceeding the MIC, or the area under the curve (AUC) of the drug concentration as a function of time. The bactericidal effect of the drug depends either on the ratio of C_max_ and MIC concentrations of the causative agent or on the time of exceeding the MIC of the drug in plasma. Doses used for the treatment of mycobacterial diseases are already known to be close to maximum tolerability, and efficacy indices are low compared to other pathogens. Only a minor part of patients attain the desired pharmacodynamic value to MIC ratios [98].

The main group of drugs against MAC infections are macrolides, which belong to the “time-dependent” antibacterial drugs, i.e., whose action depends on the time of exceeding the drug concentration over the MIC. Thus, for macrolides the elimination or killing rate becomes most important, which in turn is proportional to the bacterial growth rate [99]. An insufficient killing rate leads to the selection of cells with slower metabolism or drug-tolerant fraction. This is equivalent to increasing interim resistance, and also the surviving part of the pathogen could serve as a pool for the selection of resistant forms [100]. The growth rates of *M. tuberculosis* and *M. avium* are not so different, with one division occurring in about 23 h and 16 h, respectively [101,102,103]. The MIC distributions of clarithromycin are likely also similar (Table S2), so the critical concentrations of macrolides against *M. tuberculosis* and *M. avium* are expected to be quite close; however, macrolides are not considered effective in tuberculosis therapy, but are among the drugs of choice in the therapy of MAC disease. Since the clarithromycin susceptibility of *M. tuberculosis* obtained by the microdilution method is insufficient, it is still an open question whether macrolides are effective against tuberculosis, or if there is a significant difference in killing rates between *M. tuberculosis* and *M. avium*.

Studies are currently underway to find more effective drugs and treatment schemes. Clofazimine and bedaquiline are promising drugs. The efficacy of clofazimine in the treatment of MAC infections is comparable to that of rifampicin, but is not sufficient to prevent the emergence of macrolide resistance similar to ethambutol [65]. However, retrospective studies in the Netherlands have shown a benefit of a regimen with clofazimine and intravenous amikacin compared to standard macrolide, ethambutol, and rifampicin therapy [104].

Bedaquiline, on the other hand, shows low in vitro inhibitory concentrations against MAC pathogens, comparable to those for *M. tuberculosis* [105,106,107,108], which gives hope that it will be effective. However, for a small number of cases, the bimodal distribution of bedaquiline MICs was recorded with an increase at high (2–8 mg/L) concentrations [109,110], which indicates the existence of intrinsically resistant strains.

## 5. Conclusions

In conclusion, the poor convergence of the MIC distributions obtained in different studies and performed according to the same protocol should be noted first. Second, for *Mycobacterium avium complex* isolates, MICs of all drugs are rather high, which does not allow for effective elimination of infection due to a low ratio of drug concentration in the site of infection and MIC of the pathogen. In addition to the standardization of phenotypic methods, future validation of resistance criteria for non-tuberculosis infections should include MIC data, pK/pD, and clinical outcomes of therapy. The role of molecular methods for genotyping of pathogens and identification of resistance determinants for the development of treatment regimens is also indisputable.

## Figures and Tables

**Table 1:** Source of MIC data used in the review.

Study	PMID or ID	Method	Max Number of Isolates Tested		Reference
			*M. avium*	*M. intracellulare*	
EUCAST (10 October 2022)	-		1271	399	[27]
Lin, 2022	35804298	SLOMYCO	13	81	[28]
Ying, 2022	biorXiv DOI: 10.1101/2022.05.03.490561	SLOMYCO	24	122	[29]
Umpeleva, 2022	DOI:10.36488/cmac.2022.2.147-154	SLOMYCO	33	34	[30]
Yu, 2021	34785916	CLSI	41	48	[31]
Jaffré, 2020	32140138	SLOMYCO	80	40	[32]
Andrews, 2020	NA	SLOMYCO	212	50	[33]
Litvinov, 2018	30222736	SLOMYCO	161	16	[34]
Kwon, 2018	30012759	SLOMYCO	126	148	[35]
Maurer, 2019	29906595	SLOMYCO	333	77	[36]
Cho, 2018	29223615	CLSI	1006	823	[37]
Renvoisé, 2014	25274991	SLOMYCO	186	154	[38]
Zhao, 2014	25131955	CLSI		52	[39]
Inagaki, 2011	21393190	CLSI	167	78	[40]
Cavusoglu, 2007	18080676	CLSI	5	8	[41]
Kobashi, 2006	16944258	In-house	30	22	[42]

## Data Availability

Data is contained within the article or Supplementary Material.

## Supplementary Materials

The following are available online at https://www.mdpi.com/article/10.3390/antibiotics11121756/s1, Table S1: Studies taken into the review, Table S2: MIC distributions for clarithromycin in clinical isolates, Table S3: MIC distributions for ethambutol in clinical isolates, Table S4: MIC distributions for rifampicin in clinical isolates, Table S5: MIC distributions for rifabutin in clinical isolates, Table S6: MIC distributions for moxifloxacin in clinical isolates, Table S7: MIC distributions for ciprofloxacin in clinical isolates, Table S8: MIC distributions for isoniazid in clinical isolates, Table S9: MIC distributions for amikacin in clinical isolates, Table S10: MIC distributions for linezolid in clinical isolates, Table S11: MIC distributions for trimethoprim/sulfamethoxazole in clinical isolates, Table S12: MIC distributions for streptomycin in clinical isolates, Table S13: MIC distributions for ethionamide in clinical isolates, Table S14: MIC distributions for doxycycline in clinical isolates.
